# Lumbosacral Endoscopic Ventral–Dorsal Rhizotomy: A Novel Approach for Tone Reduction

**DOI:** 10.3390/brainsci15101030

**Published:** 2025-09-23

**Authors:** Lucinda T. Chiu, Benjamin E. Weiss, Nathan Pertsch, Olivia Rogers, Benjamin Katholi, Jeffrey S. Raskin

**Affiliations:** 1 Division of Pediatric Neurosurgery, Ann and Robert H. Lurie Children’s Hospital of Chicago, Chicago, IL 60611, USAbenjamin.weiss@northwestern.edu (B.E.W.); 2 Department of Neurological Surgery, Rush University Medical Center, Chicago, IL 60612, USA; 3 Northwestern University Feinberg Medical School, Chicago, IL 60611, USA; 4 Globus Medical, Inc., Audubon, PA 19403, USA; 5 Department of Physiatry, Shirley Ryan Ability Lab, Chicago, IL 60611, USA; 6 Department of Neurosurgery, Northwestern University Feinberg School of Medicine, Chicago, IL 60611, USA

**Keywords:** endoscopic rhizotomy, cerebral palsy, minimally invasive surgery, functional neurosurgery, pediatric

## Abstract

**Objective**: Neurosurgical interventions for medically refractory hypertonia (MRH) benefit both patients and their caregivers. Concurrent severe rotatory scoliosis and fusion constructs can make traditional microsurgical rhizotomy and navigated radiofrequency ablation (RFA) peripheral rhizotomy technically infeasible. We report the first case series of lumbosacral endoscopic ventral–dorsal rhizotomy (eVDR) in patients with MRH, and highlight this novel, minimally invasive, safe, and effective technique. **Material and Methods**: We retrospectively reviewed our single institution series of four patients with advanced hypertonia, gross motor function classification scale (GMFCS) 5, and severe rotatory scoliosis who underwent an eVDR using a flexible endoscope. We report demographics, operative characteristics, and outcomes. **Results**: Four patients underwent bilateral L1-S1 eVDR. Two patients had spastic quadriplegia and two had mixed spastic and dystonic hypertonia. Mean operative time was 225 ± 11 min and mean estimated blood loss (EBL) was 28.8 ± 26.2 mLs. Average length of stay was 2.75 days (range = 1–5 days), and average follow-up was 5.75 months (range = 3–9 months). All patients had significant decrease in bilateral lower extremity modified Ashworth Scale (mAS) scores (median decrease = 3, interquartile range [IQR] = 1; Wilcoxon rank-sum test z = −2.3, *p* = 0.02). The median decrease in Barry–Albright Dystonia Scale (BADS) scores for both patients with dystonia was 8 (IQR = 0). Two patients had minor perioperative events; none required additional surgery. All parents reported improvement in caregiving metrics. **Conclusions**: eVDR offers a safe and effective approach for tone reduction in patients with MRH and severe rotatory scoliosis and/or fusion hardware, which disallows traditional approaches.

## 1. Introduction

Medically refractory hypertonia (MRH) negatively impacts the quality of life for both patients and their caregivers. Cerebral palsy (CP) occurs with an estimated prevalence of 2.11 per 1000 live births worldwide [1]. When analyzed further, however, the prevalence differs greatly between high-income countries (1.6 per 1000 live births), low-income countries (~3.3 per 1000 live births), and the prevalence is higher than average in the United States (2.9 per 1000 live births) [2,3]. CP is an important cause of major motor impairment in childhood, with 83–88% of patients suffering from spasticity [4]. Patients with neurogenetic disorders like spinal muscular atrophy (SMA), an autosomal recessive disease occurring in 1–2 per 100,000 live births worldwide, also manifest with hypertonia-like spastic quadriplegia which progresses to limb contractures [5].

Patients with MRH suffer from multiple comorbidities, including commonly, rotatory kyphoscoliosis. About 20–30% of patients with CP have neuromuscular scoliosis, with curves beginning at an earlier age than those with idiopathic scoliosis, and progressing beyond skeletal maturity [5,6,7]. Severity of scoliosis in CP patients increases proportionally with motor disability, with GMFCS V patients being up to 23 times more likely to develop scoliosis than GMFCS level I [8].

Treatment algorithms include medication and bracing options prior to and along with surgical interventions including intrathecal baclofen pumps (ITBP), deep brain stimulation (DBS), and/or rhizotomy. Level I evidence supports selective dorsal rhizotomy (SDR) to be effective for decreasing diplegic spasticity; however, SDR can exacerbate dystonia in patients with mixed hypertonia [9]. VDR, a technique pioneered by Albright and Tyler-Kabara, provides a surgical treatment option for those patients with mixed MRH in their extremities [9,10].

Open microsurgical VDR has uncommon but important surgical limitations. Scoliosis is often mentioned in the rhizotomy literature as a potential risk of undergoing SDR, particularly in the absence of laminoplasty [11,12]. The frequency with which VDR is performed on patients with existing scoliosis who have already undergone a spinal fusion is not well studied, but it does increase the difficulty in surgical access during VDR [9,13]. The spinal hardware and heterotopic ossification can make open VDR impractical. For these patients, prior studies have considered navigated radiofrequency ablation (RFA) of the lumbosacral nerve roots at the neuroforamen to be a safe and effective alternative [14,15].

Occasionally, patients present with mixed MRH and need a lumbosacral VDR but have rotatory scoliosis so severe that neither open microsurgical nor navigated RFA VDR are feasible. The surgical approach for these afflicted patients will benefit from eVDR through a heterotopic osteotomy [16]. We present a case series of four patients with severe rotatory kyphoscoliosis who underwent lumbosacral eVDR for the management of their MRH.

## 2. Materials and Methods

### 2.1. Data Collection and Outcome Measures

This single institution, single surgeon, retrospective case series includes four young adult patients with MRH who underwent an eVDR from 2024–2025. Severe pre-operative rotatory kyphoscoliosis in all patients prohibited other approaches (e.g., open VDR and navigated RFA). Additional neurosurgical treatment options including ITBP and DBS were discussed with the CP patients and were either declined by the parents or were already present in the patient. All cases were reviewed at the Shirley Ryan AbilityLab-Lurie Children’s Hospital Complex Movement Disorder Program multi-disciplinary conference and recommended to undergo eVDR.

Patient demographics, operative characteristics, and surgical outcomes are presented. Primary outcome measures include perioperative events and tone control. Tone control severity scales using the mAS and BADS were measured by licensed physiatrists and neurosurgeons pre- and post-operatively.

Secondary outcome measures included subjective patient and caregiver reports of quality of life improvement, including with activities of daily living (ADL) and pain reduction.

**Figure 1:**
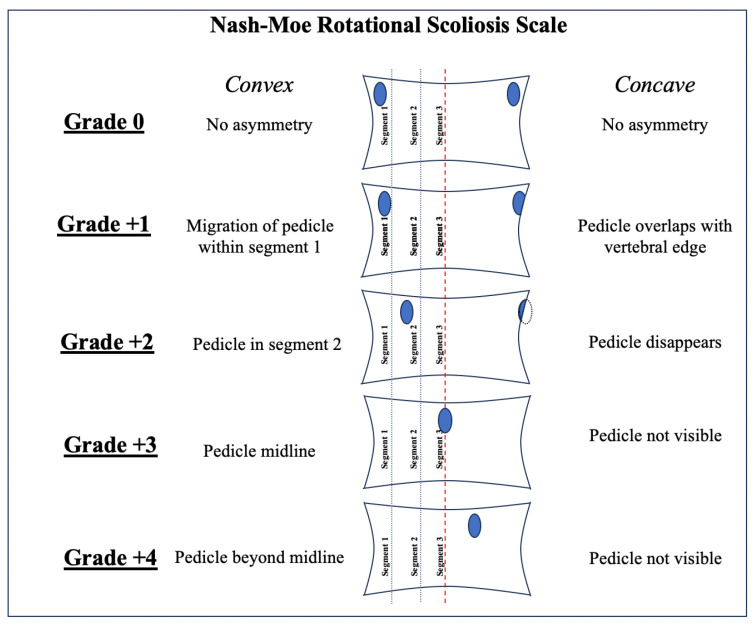
Diagram showing the Nash-Moe method of grading vertebral rotation. Each vertebral body (VB) as visualized on anterior–posterior radiographs is shown. Each VB is bisected with a midline (red dashed line). The VB on the convex side is then divided into thirds (dashed blue lines, Segment 1–3). The extent of VB rotation is determined by the position of the convex-side pedicle (dark blue oval) with relation to the above-mentioned segments, and by the visibility of the concave-side pedicle. Grade 0 (neutral position) through Grade +4 (convex-side pedicle beyond midline, concave-side pedicle not visible) are shown.

The presence of clinically relevant rotatory kyphoscoliosis was defined by a Cobb angle > 40°, as reported by a radiologist from imaging, or measured by the authors [17,18]. The Nash–Moe method was also used to assess vertebral axial rotation. This method evaluates pedicle shadow offset on anterior–posterior radiograph images to approximate vertebral body rotation on a scale of 0 to +4 (Figure 1) [19,20,21].

This study was conducted in accordance with the Declaration of Helsinki, and was deemed exempt by the Institutional Review Board Lurie Children’s Hospital (protocol code STUDY00000232 on 19 March 2025). HIPAA authorization was waived. All patients and their families were counseled on the risks of surgery, including the use of a novel approach and its rationale, and all agreed to the proposed intervention and use of data for research purposes.

### 2.2. Statistical Analysis

Tone severity scores before and after eVDR were analyzed with descriptive statistics, and using Wilcoxon rank-sum tests due to small sample size and non-normal distribution. A *p*-value < 0.05 was considered significant.

### 2.3. Surgical Procedure

**Figure 2:**
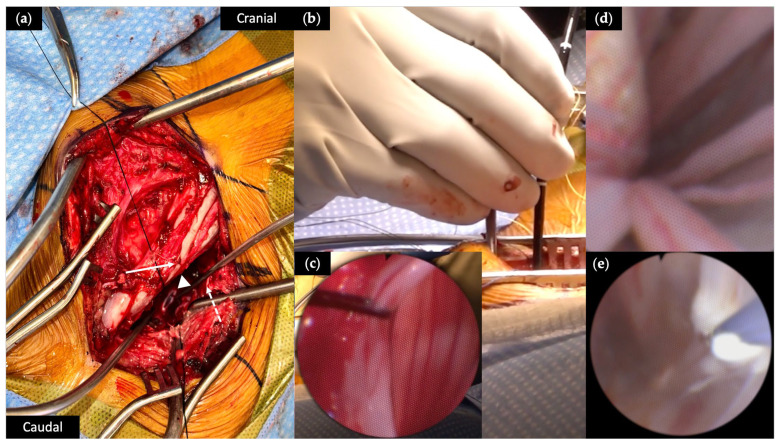
Intra-operative images of the surgical approach for endoscopic rhizotomy. (**a**) The skin incision is seen in the center of the image extending from cranial (top right) to caudal (bottom left). Paraspinal dissection reveals the patient’s left (solid arrow) and right (dashed arrow) rib cages, severely rotated off midline secondary to the patient’s rotatory scoliosis, resulting in abnormal anatomy. The dural opening (arrow head) for the endoscope is visualized deep to the ribs, with the dural edges retracted by 4-0 Nurolon sutures. (**b**,**c**) Insertion of the flexible endoscope through the dural opening. (**d**) Endoscopic view looking cephalad at the lateral spinal cord and exiting lumbosacral nerve roots. (**e**) Endoscopic view of lesioning performed with Bugbee electrocautery (Olympus America, Center Valley, PA, USA), with color change associated with successful lesioning of the target nerve root.

Our surgical technique for performing eVDR has been previously described [16]. Following pre-operative antibiotics and general endotracheal anesthesia, patients were positioned prone. Intramuscular electrodes were placed in the bilateral lower extremities to allow for intra-operative electromyography (EMG) monitoring. Planned incisions were identified using fluoroscopy. After a team pause, the skin incision was made and a paraspinal muscle dissection was performed until bony and hardware landmarks were identified. A heterotopic osteotomy of the target level was drilled as previously described [22]. Hemostasis was obtained, a dural opening was made, and the dural edges were retracted with 4-0 Nurolon suture (Figure 2a). The flexible neuro-endoscope (Karl Storz, Tuttlingen, Germany) was introduced into the cerebrospinal fluid (CSF) space (Figure 2b,c). Using continuous irrigation to promote visualization, the scope was navigated to the most inferior aspect of the intradural space. The scope was then adjusted until nerve roots were visualized as they exit the spinal column (Figure 2d). Mechanical stimulation of the nerve roots with the endoscope tip and free running EMG was used to identify the appropriate myotome. If clear stimulation of the muscle groups did not occur, nerve root levels were confirmed using fluoroscopy. Individual mixed nerve roots from lumbar 1 (L1) to sacral 1 (S1), starting with S1, were thermally lesioned with the Bugbee electrocautery (Olympus America, Center Valley, PA, USA) set to coagulation setting 10 for five to ten seconds. Extent of ablation (EoA) was volumetrically assessed following color change (Figure 2e). We irrigated until the fluid was clear, removed the endoscope, and closed the dura in a watertight fashion. We irrigated with vancomycin irrigation and placed vancomycin powder into the wound before closing the wound in a typical layered fashion. Patients were admitted to the ward or the ICU post-operatively for observation, depending on their comorbidities.

## 3. Case Presentations

### 3.1. Patient Demographics

Patient demographics are described in Table 1. Four GMFCS V patients (one female) with MRH had an average age at surgery of 20.5 ± 1.73 years. Two patients had spastic quadriplegia, and two had mixed MRH due to cerebral palsy ([CP], *n* = 3) or spinal muscular atrophy (SMA) type 2 (*n* = 1). All patients had a gastrostomy tube (G-tube) and two patients had a tracheostomy tube, one of which was ventilator-dependent. Average pre-surgical body mass index (BMI) was 18.28 ± 2.35. Prior tone control surgery included DBS (*n* = 1), and a bolus ITB test dose (*n* = 1).

**Figure 3:**
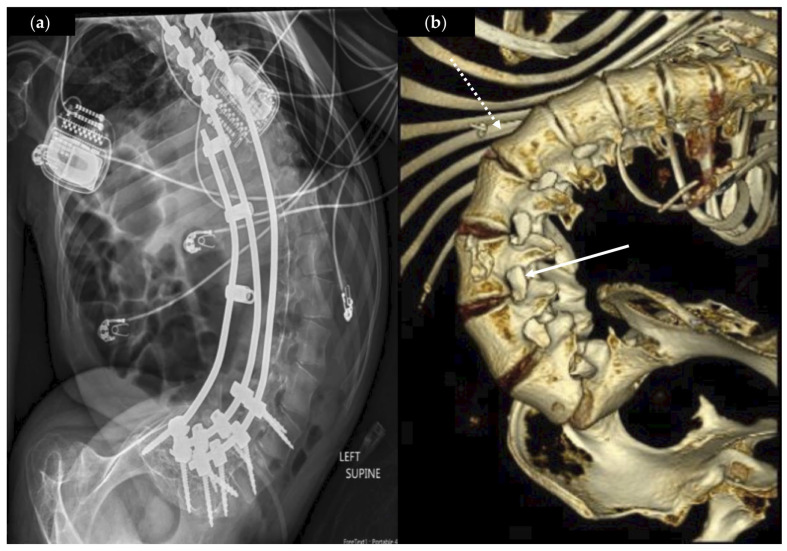
Lumbar spine radiographic images for two eVDR patients pre-operatively. (**a**) Anterior–posterior (AP) view abdominal X-ray of Patient No. 4, illustrating a long spinal fusion construct, severe rotatory scoliosis (lumbar Cobb angle 82 degrees, Nash–Moe 4+), and bilateral deep brain stimulator implantable pulse generators. (**b**) Three-dimensional reconstruction of a computed tomography (CT) lumbar spine of Patient No. 3 showing a S-shaped scoliosis (lumbar Cobb angle 143 degrees) with a severe rotational component (Nash-Moe 4+). The concave (solid arrow) and convex (dashed arrow) sides of the scoliosis curve with difference in visualization of the neural foramen are also illustrated.

All patients had clinically relevant, severe rotatory kyphoscoliosis with an average Cobb angle of 85.1 ± 40.6° (Figure 3a), and three patients had a Nash–Moe index of +4, with pedicles rotated beyond midline of the vertebral body (Figure 3b). The majority (*n* = 3) of patients had a history of thoracolumbar posterior spinal fusion.

### 3.2. Operative Characteristics

All patients underwent a bilateral L1-S1 eVDR, and one patient also had a left T12 eVDR targeting left hip pain. Mean operative time was 225 ± 11 min and mean EBL was 28.8 ± 26.2 mL. Average length of stay was 2.75 days (range 1–5 days; Table 2). Mean follow-up duration was 5.75 months (range 3–9 months; Table 2).

### 3.3. Surgical Outcomes

**Figure 4:**
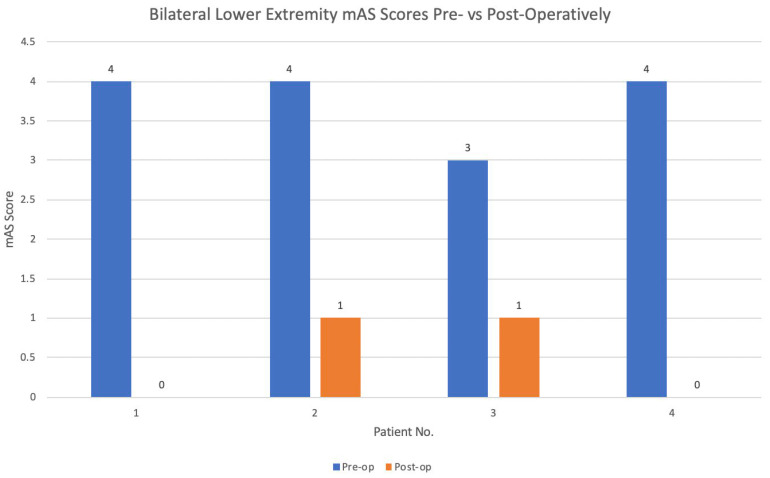
Bar graph of bilateral lower extremity mAS scores before and after eVDR at last follow-up. Wilcoxon rank-sum test comparing pre- and post-operative BLE mAS scores: BLE mAS z = −2.3, *p* = 0.02.

All four patients experienced post-operative improvement in their bilateral lower extremity (BLE) tone following eVDR compared to their pre-operative baseline (Figure 4). BADS and mAS scores remained improved at last follow-up. The median change in BLE mAS score was 3.5. Using the Wilcoxon rank-sum test, there was a statistically significant change in BLE mAS scores following eVDR for all patients (z = −2.31, *p* = 0.02). No significant change was found for BUE mAS scores (z = −1.15, *p* = 0.25). In the two patients with dystonia, the overall BADS score decreased by 8 (IQR = 0, Case No. 1 pre-op BADS 25, post-op 17; Case No. 3, pre-op BADS 30, post-op 22).

At follow-up, all caregivers noted improved tone (*n* = 4) and ease with activities of daily living and caregiving including positioning (*n* = 2) and transfers (*n* = 1) (Table 2). One caregiver reported significant improvement in pain post-operatively.

### 3.4. Perioperative Events

Overall, the four patients tolerated the eVDR procedure well. No patients required reoperation. Two patients had minor perioperative events (Table 2). One patient has persistent neuropathic pain at three-month follow-up in the bilateral legs from the knees down and was started on gabapentin with improvement. One patient experienced superficial wound dehiscence at one month following surgery and was treated with a short course of enteral antibiotics and local wound care, with resolution. There was no incidence of new muscle atrophy, venous thrombosis, or new permanent urinary retention.

## 4. Discussion

While prior studies have shown encouraging benefits for non-selective lumbosacral VDR with an open approach, the use of eVDR has been scarcely reported [16]. While limited by small sample size, this study is the first retrospective case series that reports on the feasibility and promising efficacy of eVDR for patients with lower extremity MRH and limited alternative treatment options due to their rotatory scoliosis and fusion mass.

### 4.1. Safety and Indications of eVDR

While eVDR is a novel technique, the principle approaches and surgical corridors it utilizes have been previously used and studied in the treatment of patients with hypertonia and scoliosis with or without spinal fusion [22,23].

Patients with MRH benefit from both nonselective lumbosacral VDR and ITBP, although VDR obviates the relative contraindications of ITBP including scoliosis, epilepsy, and low BMI [24,25,26]. Our patient cohort has low BMI, severe rotational scoliosis, and epilepsy.

BMI presents surgical challenges for lumbosacral SDR and ITBP implantation and can be correlated with post-operative complications including wound infections. Average BMI for our patients was 18.28, with one patient having a BMI as low as 14.8. While one of our patients had a superficial SSI not requiring surgery (BMI = 19.1), larger studies are needed to understand the relation of these patient factors with those undergoing VDR. One retrospective study of patients undergoing SDR found obesity (BMI z-score ≥ 1.64) to be predictive of prolonged wound healing and surgical site infection (SSI) [27]. Low BMI, on the other hand, has not been found to be strongly associated with infection following ITBP implantation, despite the concern of erosion through the minimal subcutaneous fat in these patients [28,29,30,31,32]. Weight gain following SDR has been described, but the association between pre-operative BMI and SDR or VDR remains unknown [33].

Intradural access is difficult in patients with a spinal fusion, as the operative corridor is limited by both spinal instrumentation and heterotopic ossification. Severe rotatory scoliosis makes this anatomically abnormal corridor even more constrained. Heterotopic osteotomy for intrathecal access in patients with spinal fusion has been previously described for both ITB bolus test dose and placement of an ITBP [22,34]. Unopposed CSF egress upon needle withdrawal is reported and may be due to the lack of typical anatomical structures; this mechanism may mediate a higher rate of post-dural puncture headache [22]. Our case series was not complicated by CSF fistula, potentially due to a proper dural closure compared with the previously reported dural puncture technique.

Endoscopic spine surgery for adult degenerative spine disease, in some cohort studies, outperforms open microsurgical techniques with decreased tissue damage, decreased post-operative pain medication needs, reduced blood loss and operative times, and shorter hospital stays [35,36,37]. In children, endoscopic intradural surgery for arachnoid cyst fenestration and focal rhizotomy has been performed [23,38,39]. Our case series reports a mean operative time of 225 min and a mean EBL of 30 cc, with no required perioperative blood product transfusions; these results are comparable to those published for microsurgical VDR in a similar demographic [9]. The length of stay for eVDR is shorter than microsurgical VDR (2.8 days versus 7.8 days, respectively) and there were fewer wound healing complications (one versus three cases), none requiring revision. The extent of dissection to facilitate the operative corridor may have contributed to the shorter length of stay (e.g., limited paraspinal dissection, and limited osteotomy).

Pain control following VDR procedures contributes to an improved outcome and allows for early rehabilitation. One of four patients has persistent bilateral lower extremity neuropathic pain requiring gabapentin 100 mg every 8 h. Pain following rhizotomy procedures is largely focused on SDR, where the primary pain generators are believed to be from bony work, nerve root manipulation, and/or muscle spasms in the extremities [40,41]. Neuropathic pain and sensory changes following SDR have been reported in 5–14% of patients, with 4–6.5% reporting persistent sensory changes [42,43,44]. Pre-operative gabapentin has been incorporated into some hospital SDR protocols [42]. Though the exact rate of persistent neuropathic pain following eVDR has yet to be studied, transient pain is expected, and treatment with gabapentin is effective.

### 4.2. Efficacy of eVDR

Microsurgical lumbosacral VDR is effective for tone control and improving quality of life in patients with MRH [9]. Our eVDR cohort of four patients also showed a statistically significant reduction in BLE mAS score by an average of 3.5. Within the adult stroke literature, a 1-point change in mAS score has been suggested to have clinical significance [45]. Patients with quadriplegic hypertonia who undergo lumbosacral SDR are reported in multiple studies to have suprasegmental changes in motor activation, with decreased mAS scores in the upper extremities (range = 0–2.9) through a yet unknown mechanism [46]. While no statistically significant improvement in upper-extremity mAS was seen post-operatively, every capable patient and all caregivers reported post-operative improvement in ease of ADLs. Statistical significance was unable to be calculated for overall BADS scores given such a small sample size, and the minimum clinical important difference for BADS remains unknown. The objective tone reduction and subjective quality of life improvement findings are encouraging, similar to reported microsurgical VDR approaches, and warrant further study.

Disease etiology of the patient’s MRH (SMA versus CP) may also have influenced surgical outcomes, though the underlying mechanism and exact pathophysiology is unknown given the current paucity of studies on VDR for SMA patients with hypertonia, and the small sample size (*n* = 1 with SMA) within this cohort.

### 4.3. Technical Challenges and Surgical Considerations

Endoscopic VDR facilitates a neurosurgery technique through a minimal access approach to achieve similar tone reduction for patients suffering from MRH. From our initial experience in this small case series, endoscopic VDR has hardware-, patient- and technique-related challenges.

The flexible endoscope has one working port through which an instrument or irrigation can effectively flow. There is currently no bipolar stimulator or other tool to reliably identify nerve roots. The flexible endoscope is limited to 90 degrees at the tip, and this may require cephalad and caudad approaches to reach every nerve root in certain spine morphologies.

We chose eVDR for patients with severe rotatory scoliosis and spinal fusion (75% of our patients) preventing us from performing microsurgical VDR or RFA. This anatomy makes localization of the neural foramen and nerve roots challenging, often requiring adjunctive fluoroscopy and bed rotation to identify nerve roots. We found that a cephalad to caudad approach improved the ability to activate and identify nerve roots, and that exiting nerve roots on the concave side were easier to identify than those hidden under the convex side of the curve (Figure 3b).

Maintaining visualization requires continuous irrigation. The ability to perform nerve root dissection and to measure extent of lesioning as in the microsurgical VDR technique is limited using the eVDR technique as the flexible endoscope used has only one working channel and no ability to dissect the nerve root.

All patients in this cohort were carefully selected following multi-disciplinary discussion. This procedure was offered as an alternative to no surgical option, and we found it to be safe and effective for MRH. Thorough and careful patient selection for eVDR is critical.

### 4.4. Study Limitations

This study is retrospective and includes a small sample size of patients from a single institution who underwent an eVDR with a single surgeon. Thus, conclusions from this study may be limited in their generalizability. Future research directions include cost analysis of eVDR compared to microsurgical techniques, and the inclusion of more objective quality of life metrics. Follow-up is limited. Larger, prospective, multi-center studies with the use of a control group and longer follow-up (>12 months) on this novel approach in properly selected patients are warranted.

## 5. Conclusions

Endoscopic VDR may offer a safe, innovative, and minimal access surgical option for patients with MRH and limited surgical alternatives due to concurrent severe rotatory scoliosis and spinal fusion. Further studies are necessary to establish eVDR as an additional surgical technique within the functional neurosurgery toolkit for these patients.

## Figures and Tables

**Table 1:** Patient Demographics.

Case No.	Age (Years), Sex	Race/ Ethnicity	Dominant Movement Disorder	Diagnosis Etiology	GMFCS (I–V)	Trach?	G-Tube?	BMI	Scoliosis, Cobb (°), Curve	Scoliosis, Nash-Moe	Prior Spinal Fusion	Prior Functional Surgery
1	22, M	Asian	Quadriplegia, mixed	CP (HIE)	V	Y	Y	19.9	T10-L4, 51.5 L	+2, L3	Y	Y, bolus ITB test dose
2	21, M	Hispanic	Quadriplegia, spastic	CP (unknown)	V	N	Y	19.3	T7-L4, 64 D	+4, L3	Y	N
3	21, F	Hispanic	Quadriplegia, spastic	SMA2	V	Y *	Y	14.8	T12-S1, 143 D ^	+4, T12	N	N
4	18, M	Hispanic	Quadriplegia, mixed	CP (pre-term)	V	N	Y	19.1	T9-L5, 82 L	+4, T12	Y	Y, DBS

M, male; F, female; GMFCS, gross motor function classification scale; G-tube, gastrostomy tube; Trach, tracheostomy; BMI, body mass index; CP, cerebral palsy; HIE, hypoxic–ischemic encephalopathy; SMA2, spinal muscular atrophy type 2; D, dextroscoliosis; L, levoscoliosis; ITB, intrathecal baclofen; DBS, deep brain stimulator. * Ventilator-dependent. ^ S-shaped scoliosis, dextroscoliosis in lumbar region.

**Table 2:** Operative Characteristics and Perioperative Events.

Case No.	eVDR Procedure Levels	Access Laminotomy Level	Operative Time (Minutes)	EBL (mLs)	LOS (Days)	Follow-Up (Months)	Perioperative Events?	Caregiving Improvement
1	Left T12, bilateral L1-S1	L3	233	50	2	8	No	Transfers
2	Bilateral L1-S1	L3	230	<5	5	9	No	Positioning
3	Bilateral L1-S1	T12	209	50	3	3	Mild neuropathic pain	Improved ROM
4	Bilateral L1-S1	T12-L1	229	10	1	3	Superficial wound dehiscence	Positioning

eVDR, endoscopic ventral dorsal rhizotomy; EBL, estimated blood loss; mLs, milliliters; LOS, length of stay; T, thoracic; L, lumbar; S, sacral; ROM, range of motion.

## Data Availability

All necessary data are included within the article.

## Abbreviations

The following abbreviations are used in this manuscript:

CP	Cerebral palsy
SMA	Spinal muscular atrophy
eVDR	Endoscopic ventral dorsal rhizotomy
SDR	Selective dorsal rhizotomy
RFA	Radiofrequency ablation
mAS	Modified Ashworth Scale
BADS	Barry–Albright Dystonia Scale
GMFCS	Gross motor function classification score
ITB	Intrathecal baclofen
CSF	Cerebrospinal fluid
DBS	Deep brain stimulator
SSI	Surgical site infection
